# Differences in Serum Levels of Magnesium, Phosphate, and Albumin for HAART-Experienced and HAART-Naïve Female Patients Attending Parirenyatwa Opportunistic Infections Clinic in Harare, Zimbabwe

**DOI:** 10.1155/2013/383214

**Published:** 2013-09-04

**Authors:** Denise Mudzinge, Tinashe Kenny Nyazika, Tawanda Jonathan Chisango, Danai Tavonga Zhou

**Affiliations:** ^1^Department of Medical Laboratory Sciences, College of Health Sciences, University of Zimbabwe, P.O. Box A 178, Harare, Zimbabwe; ^2^Department of Chemical Pathology, College of Health Sciences, University of Zimbabwe, P.O. Box A 178, Harare, Zimbabwe; ^3^Biotechnology Department, Chinhoyi University of Technology, P.O. Box 2274, Chinhoyi, Zimbabwe

## Abstract

Antiretroviral therapy inhibits HIV replication, maintains health, and preserves life. However, both antiretroviral therapy and HIV infection have been reported to have short- and long-term effects on bone metabolism. A cross-sectional study was performed to compare serum bone profiles in HIV positive patients on highly active antiretroviral therapy and compare them to therapy-naïve patients. Serum levels of calcium, magnesium, phosphate, and albumin were measured in 40 female participants on highly active antiretroviral therapy, recruited sequentially from Parirenyatwa Opportunistic Infections Clinic, Harare, Zimbabwe. The 40 women were matched for age with 40 antiretroviral therapy-naïve women. Magnesium, phosphate, and albumin levels were significantly higher in the therapy-naïve than in therapy-experienced patients. There was no statistically significant difference in calcium levels of the two groups of women. Evidence from this study suggests that highly active antiretroviral therapy lowers levels of magnesium, phosphate, and albumin but has no effect on levels of serum calcium.

## 1. Introduction

Zimbabwe is in sub-Saharan Africa which is at the epicenter of the human immune deficiency virus (HIV) epidemic. According to UNAIDS the prevalence of HIV in Zimbabwe has decreased to about 1 in 10 adults (2012) from a high one of almost 1 in 4 in 2002 [1]. While the decline is commendable HIV infection still remains a major problem in Zimbabwe with 14.3% of adults being HIV positive [2]. 

The high disease burden of HIV has necessitated a rapid increase in the use of highly active antiretroviral therapy (HAART). As of 2012, over 476 thousand HIV-infected Zimbabweans were on HAART compared to only 8000 in 2003 [1]. In the Zimbabwe National Program, first line drug combinations include a dual combination of tenofovir/TDF/disoproxil fumarate (Gilead Sciences, USA); a nucleoside reverse transcriptase inhibitor (NRTI) and lamivudine/3TC/2,3 dideoxy-3-thiacytidine (GlaxoSmithKline and Pfizer, UK), an NRTI and a triple combination of tenofovir, lamivudine with nevirapine/XR/viramune (Boehringer Ingelheim, USA) a non-nucleoside reverse Transcriptase inhibitor (NNRTI) [3, 4]. In the event of treatment failure the patients are treated with second line drugs including zidovudine/AZT (Company) an NRTI; didanosine/DDI/Videx (Bristol-Myers Squibb Co, USA), an NRTI together with lopinavir and ritonavir; protease inhibitors (PIs) also known as kaletra/aluvia (Abbot Laboratories) [3]. 

HAART has reduced both the morbidity and mortality of HIV-infected people due to AIDS. However HAART is reported to have adverse side effects, one of which is bone mineralization. Changes in serum levels of biochemical markers of bone metabolism have been shown elsewhere in experimental and clinical studies [5–7]. A number of studies from various parts of the world have reported that HAART may have effects on bone metabolism via vitamin D [8] and/or parathyroid hormone [9]. This has been linked to the greater risk of developing fractures in HIV-infected patients on HAART. Evidence has also been shown in some studies linking current use of HAART to low levels of vitamin D: calcidiol (25-hydroxycholecalciferol) [10]. Calcidiol is most commonly determined in measurements of vitamin D status because of its longer half-life than the active form of vitamin D: calcitriol (1,25 cholecalciferol) [11].

Osteopenia and osteoporosis are conditions involving the weakening of bones which differ in the degree to which bones weaken [12]. Osteopenia and osteoporosis are common manifestations in HAART-experienced patients and are both due to low bone mineral density [13]. In a recent study of a large number of HIV-infected participants, 53.7% of the patients had osteopenia whilst 26.8% had osteoporosis [14, 15]. WHO recommends use of bone mineral density as a marker of bone disorders like osteopenia and osteoporosis [16]. Phosphorus, calcium, and magnesium have a role in bone metabolism. Calcium and phosphate combine together to form hydroxyapatite the inorganic part of bone. Magnesium is an intracellular component of bone cells [17]. A number of studies such as the one by De Socio et al. have reported changes in bone density accompanied by changes in serum levels of phosphate and alkaline phosphate in HIV-infected participants before and after antiretroviral therapy [18, 19].

A European study showed that Tenofovir increases bone turnover and decreases bone mineral density in treated HIV-infected adults [20]. Results in a US study showed that protease inhibitors increase the incidence of osteopenia and osteoporosis in men [21]. 

Tenofovir, a drug recently introduced to Zimbabwe's First line treatment, has been associated with osteomalacia, low serum levels of phosphate, and elevated alkaline phosphate in a Swiss study [22]. A Nigerian study provided evidence for an elevation in albumin levels due to HAART [6]. This is in contradiction to Southern India study that showed that 84% of patients on ART had low levels of albumin (<28 g/L) as compared to ART naïve patients [23].

There is no published evidence for the bone effect of prescribed first and second lines HAART regimens on Zimbabweans. The objective of this study was to provide evidence of changes in serum bone profiles, if any, in a Zimbabwean population of women using currently prescribed HAART regimens and compare them to a similar group of HIV-infected women who are not yet on HAART (Table 2). The broad objective was to find a noninvasive and cheap method of detecting changes in bone metabolism in HIV-infected and HAART-experienced patients in our resource-limited settings.

## 2. Materials and Methods

### 2.1. Study Site

Parirenyatwa Group of Hospitals is a group of public and teaching hospitals found in the capital city of Zimbabwe. The group of hospitals are referral hospitals servicing casualties from Harare City Centre and its environs, together with referred cases from a number of clinics around Harare and other Zimbabwean provinces and districts. The clientele attending the Parirenyatwa Opportunistic Infections (OI) Clinic, the site of our study, is diverse in terms of geographical location, but many are from low-income groups. Those participants who were on HAART were taking a combination of tenofovir, lamivudine and nevirapine for the first line treatment and zidovudine, didanosine, and lopinavir/ritonavir for second line treatment. We sequentially recruited 40 HIV-infected women, aged 18 to 40 years who had been on first line HAART for more than 6 months and consented to be part of our study. 40 HIV-infected women who were HAART-naïve were matched for age with the 40 consenting HAART-experienced women. All the women had no evidence of critical illness at the time of the study. 

### 2.2. Ethical Considerations

Permission to work with the Parirenyatwa OI Clinic was sought from the Clinical Director of Parirenyatwa Group of Hospitals, whilst ethical clearance was given by the Joint Research Ethics Committee of the Parirenyatwa Group of Hospitals and University of Zimbabwe (JREC) after they were satisfied that the research protocol was ethically sound. Participants gave written consent after being informed fully about their role, risks, and benefits in the study and being satisfied that their identities would be protected using anonymous numerical identifiers on all documents and specimen containers. 

### 2.3. Sample Size Calculation

Consider

(1) $$\begin{array}{c} N=\left[\frac{{\left(Z_{\alpha /2}+Z_{1}-\beta \right)}^{2}}{{\left(p_{1}-p_{2}\right)}^{2}}\right]\left[p_{1}\left(1-p_{1}\right)+\left(1-p_{2}\right)\right], \end{array}$$

where *n* = minimum sample size, *z* = test statistic (at 5%), *p*_1_ = proportion with desired characteristic (0.05), *Z*_*α*/2_ = 1.96, *Z*_1_ − *β*, *p*_1_ = 0.63, *p*_2_ = 0.28,

– *n* = ((1.96+0.84)^2^/(0.63−0.84)^2^)(0.63(1 − 0.63) + 0.28(1 − 0.28)) = 27.14 = 28,
– Final *n* = 28 + Refusal Rate (Refusal Rate = 15% of *n*),
– *n* = 28 + 12.17 = 40.17.

Therefore overall minimum sample size = 80 (40 on HAART and 40 HAART-naïve).

### 2.4. Data and Sample Collection

Demographic data was obtained from the patients and clinic records (Table 1). Blood was drawn into plain tubes by a phlebotomist at the clinic during patients' routine visits. Serum was aliquoted into 2 mL cryotubes and stored at −20°C for up to 20 days. 

### 2.5. Sample Analysis

Serum samples were thawed once at room temperature, and analysis was carried out on a BS120 Mindray Chemistry analyzer. The chemistry analyzer was calibrated, and then controls and samples were assayed for total calcium, magnesium, phosphate, and albumin. Corrected calcium was calculated for participants with low albumin levels.

## 3. Results

The mean assayed values for the female patients on HAART (together with the normal reference ranges) were: total calcium 6.92 mg/dL (reference range 8.80–9.50 mg/dL), Magnesium 1.78 mg/dL (Reference Range 1.46–2.68 mg/dL), Phosphate 5.10 mg/dL (reference range 1.80–5.10 mg/dL) and Albumin 2.87 mg/dL (reference 2.80–5.30 mg/dL). The means of the HAART-naïve women were; Total calcium 8.40 mg/dL, Magnesium 2.55 mg/dL, Phosphate 5.10 mg/dL and Albumin 3.25 mg/dL. 

## 4. Conclusion

There was no significant difference between total calcium levels in patients on HAART and HAART-naïve patients. However, the values of calcium for the two groups in our study fall below the normal range. Our results suggest that use of both HIV and HAART may lead to hypocalcaemia. This contradicts with studies done in America where elevation in serum calcium levels in women on antiretroviral therapy including tenofovir was linked to an increase in bone loss [24]. Our results seem to be in agreement with a different set of studies described in India that reported lower levels of the active form of vitamin D (calcitriol), resulting in reduced intestinal calcium absorption. Their report, as we observed, described a decrease in serum calcium levels after HAART initiation [25]. It becomes essential to measure levels of vitamin D in future studies, in order to answer the important question of whether HAART affects bone metabolism via vitamin D. 

There were other interesting differences in serum bone profiles of the women in our study. Magnesium and albumin levels were within normal ranges for both groups of women, but the HAART-experienced had lower magnesium and albumin levels than the HAART-naïve patients. Our findings for magnesium do not however agree with two studies: a Brazilian study that showed hypomagnesaemia occurring in 29% of patients on antiretroviral therapy [26] and a study done in Nigeria that showed an elevation in serum albumin, months after antiretroviral therapy initiation [6]. Our results for albumin do correlate with the findings of a study carried out in Southern India, in which serum albumin levels were lower after antiretroviral therapy [23]. 

Phosphate levels were elevated in HAART-naïve patients, and their levels were above the normal range whilst the HAART-experienced women have on average phosphate levels that fall within normal ranges. Our study contradicts with studies which showed a link between use of tenofovir and hypophosphatemia [27] and studies done in America that showed a decrease in serum phosphate due to reduced renal retention [28]. 

The results of our study show both expected and unexpected results. It is important to improve this study by increasing the number of study sites, increasing the power by enlarging the sample size, and also measuring the levels of vitamin D (calcidiol or calcitriol). The results of this and future studies will be used to advocate for change in practice in treatment of HIV-infected women on HAART. Clinicians and decision makers may have to consider giving patients on HAART vitamin D supplements to avoid weakening of bones in Zimbabweans on HAART. Another study involving both men and women is also necessary in order to determine what serum bone profiles changes may be happening in men due to HIV infection and/or HAART. Is serum bone profiling a potentially noninvasive, inexpensive method that can be used routinely to detect changes in bone metabolism in the HIV infected patients in our resource-limited setting? This study shows evidence of changes in serum bone profiles. A larger study that also aims to determine association between serum bone profiles and measurement of the WHO gold standard (mineral bone density) may help answer that question.

## Figures and Tables

**Table 1:** Demographic data of 80 female participants.

Characteristic	HAART+ (*N* = 40)	HAART-naïve (*N* = 40)	*P* value, significant difference
Mean age/years	24.4 (5.0)	24.1 (5.2)	0.05, NS
Time since diagnosis of HIV/years	8.8 (2.6)	7.5 (2.6)	<0.05, significant difference
Gravidae/number of pregnancies	3.8 (0.6)	3.5 (0.6)	<0.05, significant difference
Para/number of surviving children	2.2 (1.2)	2.3 (1.2)	>0.05, NS
Income/average per month in USD	62.2 (11.1)	62.0 (10.5)	>0.05, NS

**Table 2:** Comparison of serum bone profiles of female patients on HAART and before HAART.

	HAART+	HAART-naïve	*P* value, significance
Mean total calcium (mmol/L)	6.92	8.4	
Standard deviation for mean total calcium	0.024	0.03	
Standard error for mean total calcium	0.18	0.202	
95% confidence interval	1.67	1.969	*P* = 0.23, no significant difference
Mean magnesium (mmol/L)	3.00	4.32	
Standard deviation for mean magnesium	0.021	0.029	
Standard error for mean magnesium	0.158	0.193	
95% confidence interval	0.692	0.99	*P* = 0.033, significant difference
Mean phosphate (mmol/L)	5.27	12.49	
Standard deviation for mean phosphate	0.23	0.36	
Standard error for mean phosphate	1.696	2.41	
95% confidence interval	0.865	3.31	*P* = 0.015, significant difference
Mean albumin (mg/dL)	2.87	3.24	
Standard deviation for mean albumin	0.6	0.82	
Standard error for mean albumin	4.42	5.44	
95% confidence interval	2.75	3.08	*P* = 0.023, significant difference
